# Disclosing racial trauma in psychological therapy: Exploring the experiences of racially minoritised people in the UK

**DOI:** 10.1111/papt.12592

**Published:** 2025-04-18

**Authors:** Nicole K. S. Samuel, Laura M. Simonds

**Affiliations:** ^1^ Psychologically Informed Environments Centrepoint London UK; ^2^ Department of Psychological Interventions, School of Psychology University of Surrey Guildford UK

**Keywords:** antiracism, clinical practice, equality, diversity and inclusion (EDI), psychological therapy, racially minoritised, racism, therapeutic relationship

## Abstract

**Objectives:**

Exposure to racism is repeatedly experienced by individuals from racially minoritised backgrounds, and has a range of negative emotional, physical and social consequences; however, its traumatising effects are under‐recognised. Further, psychological therapists often lack sufficient knowledge, training and confidence to sensitively manage conversations about racism. As this has important implications for the standards of care this population receives, this study explored how racially minoritised clients experience disclosing, or attempting to disclose racial trauma in psychological therapy.

**Design:**

The study utilised an online qualitative survey design.

**Methods:**

Participants were 28 adults who identified as belonging to minoritised racial groups and had engaged in psychological therapy in the UK. Therapy spanned a range of modalities, and providers included the NHS, private therapists/organisations, charities and university services. Data were analysed using thematic analysis.

**Results:**

Three superordinate themes were constructed: *The Dangers of Disclosure* *;* *Holding the Burden*; and *Feeling Heard and Held*. These demonstrated both the range of potential harms and burdens associated with disclosures of racial trauma in therapy, and examples of meaningful, validating therapist responses to disclosure.

**Conclusions:**

Therapists, regardless of racial heritage, have the potential to both perpetuate harm and provide meaningful support in response to disclosures of racial trauma. Racial reflexivity and education on racism and racial trauma are essential to ethical and antiracist therapeutic practice, and crucial to safeguarding racially minoritised clients from racial harm in therapy. These must be embedded in training, practice and policy for meaningful improvements in racially minoritised clients' experiences of therapy to occur.

## INTRODUCTION

### The impact of racism as *racial trauma*

Racism refers to ‘the normalization and legitimization of an array of dynamics … that routinely advantage whites while producing cumulative and chronic adverse outcomes for people of color’ (Lawrence & Keleher, 2004, Structural Racism: Definition). Premised on white supremacy, socially constructed systems of racialisation, racial hierarchy and whiteness have systemically afforded power, privilege and resources to those racialised as ‘white’, whilst maintaining and normalising the subjugation and minoritisation of those racialised as ‘other’ (Alleyne, 2022; Boxhill, 2023; Kinouani, 2021; Lawrence & Keleher, 2004; Race Equality Action Group, n.d.). Acts of racism go beyond overt, direct and interpersonal acts, and include covert, indirect and institutional forms, which may be committed by individuals, communities and institutions, whether intentional or otherwise (All Together Now, n.d.). Examples include, but are not limited to, racial microaggressions or ‘subtle acts of exclusion’ (SAE; Jana & Baran, 2023, SAE Defined: Anatomy of an SAE), racialised threats and attacks, police violence/harassment, hostile environment policies, workplace discrimination, medical mistreatment and increased rates of detention in prison and inpatient settings (Williams et al., 2018).

Whether experienced directly, learnt about or witnessed towards others that one identifies with, exposure to racism may confer physical and/or psychological damage to racially minoritised individuals via actual or threatened harm, injury, shame or humiliation (Carter, 2007; Comas‐Díaz et al., 2019). An emerging body of literature has highlighted the ‘cumulative traumatizing impact of racism’ (Williams et al., 2021, p. 1), resulting from the multiplicity of racist incidents experienced by racially minoritised people across the lifespan. These traumatising effects of racism and the emotional, psychological and physical reactions of racially minoritised people to painful experiences of racism have been referred to as *racial trauma* (Butts, 2002; Carter, 2007; Hemmings & Evans, 2018; Williams et al., 2018). For people marginalised by their racialisation, exposure to racism may be reminiscent of historic systems of oppression and persecution against their relatives or ancestors (Comas‐Díaz et al., 2019). In the UK, the pervasiveness of racism was recently underscored in findings that the Metropolitan Police Service is *still* institutionally racist (Casey, 2023), decades after this was highlighted in the Macpherson (1999) Report. The impact is significant: a ‘generational mistrust of the police amongst Black Londoners’ (Casey, 2023, p. 17) who are ‘over‐policed and under‐protected’ (p. 331). As this demonstrates, the impact of racist events and systems is compounded by intergenerational traumatisation, and racial trauma is felt collectively, throughout whole communities (Santiago‐Rivera et al., 2016).

### Racism, racial trauma and UK mental health systems

Although the impact of racism is well‐documented and wide‐ranging, the existence and impact of racial trauma remain underrecognised (Kinouani, 2021). This is particularly striking in the UK; at the time of writing, no empirical research on racial trauma in the UK context has been published. This is despite national recommendations calling for recognition of the effects of racism on the mental wellbeing of racially minoritised communities, made two decades ago (Department of Health [DoH], 2005). Despite assertions that ‘discrimination in the NHS is unacceptable in any form’ (DoH, 2005, p.41), an abundance of literature demonstrates the ongoing pervasiveness of racialised disparities throughout UK mental health systems. Racially minoritised people in the UK continue to experience higher rates of distress; poorer access to, outcomes from, and experiences of, psychological interventions; and exposure to coercive ‘treatment’ practices, including detention in inpatient settings, which are both longer in duration and more frequent than their white counterparts' (Beck et al., 2019; Bignall et al., 2019; Fernando, 2017; Hicks & Butler, 2021; Kapadia et al., 2022; Kinouani, 2021; Mercer et al., 2019; Nazroo et al., 2020). It is unsurprising that such inequities have persisted given the lack of space afforded to racially minoritised people's voices and experiences in psychological research (Kinouani, 2021); this begets a ‘conspiracy of silence’ (p.35) which inevitably sidelines, and also reinforces, the deleterious impacts of racism on the wellbeing of racially minoritised people. This practice both demonstrates and upholds the institutionalisation of whiteness and racism in clinical research and therapeutic practice in the UK, perpetuating the well‐established cycles of inequity described above (Kinouani, 2021; Patel, 2021). In these ways, the failure of UK mental health systems to recognise the distress caused by racism is reminiscent of Britain's obfuscation of its own colonial history in the face of ongoing coloniality.

### Conceptualisation: Traumatisation versus PTSD

Underrecognition of racial trauma might also be, in part, due to how it is conceptualised. Although racial trauma often evokes sequalae that are consistent with the diagnostic entity of PTSD, such as intrusions, avoidance, hypervigilance, heightened anxiety, emotional numbing, guilt and shame (Kirkinis et al., 2018), Western diagnostic systems fail to acknowledge the traumatic impact of racism (Butts, 2002; Carter, 2007; Williams et al., 2018), which inevitably has implications for service access (Kirkinis et al., 2018). Conversely, it has been argued that locating traumatic experiences of racism within psychiatric nosology would be inappropriate given the harmful potential for this to lead to pathologisation of a person's understandable responses to racism (Kirkinis et al., 2018). A distinction has also been made in the literature between ‘race‐related stress’ and ‘racial trauma’ on the basis of whether or not a person is able to ‘cope’ with racist events or conditions. However, conceptualising this as ‘stress’ could be experienced as minimising and invalidating, and places the burden of responsibility on racially minoritised individuals, rather than targeting the oppressive structures they are subjected to (see also Shwaik, 2023). Across different definitions, Kirkinis and colleagues (2018) highlight a lack of empirical attention to indirect experiences of racism, such as intergenerational or vicarious forms. Similarly, Kira (2010, pp. 129–130) notes that the cumulative and ongoing nature of ‘collective identity’ traumas (i.e. events – like racism – which threaten the existence or interest of a social group that one identifies as belonging to) – are typically overlooked in favour of historical events and experiences, and those at the level the of individual, such as sexual violence. These critiques are addressed in the current study, wherein the umbrella term ‘racial trauma’ is used to denote the harmful impact of racism, as experienced by racially minoritised groups: (i) regardless of whether or not it meets criteria for PTSD; (ii) where the traumatic experience relates to any form of direct, indirect, vicarious, intergenerational, interpersonal, systemic or institutional racism; and (iii) whether the experience relates to an individual incident or the cumulative effects of repeated exposure to racism.

### Racial trauma and psychological therapy

Given the enduring and cumulative impacts of historic and ongoing racism, when racially minoritised people present in mental health services, a crucial aspect of effective intervention is recognising and addressing the significance of experiences of racism and the existence of racial trauma. Evidence suggests, however, that psychological therapists might avoid asking about race, ethnicity and culture for fear of ‘getting it wrong’ (Beck, 2019; Naz et al., 2019). Avoidance not only risks missing opportunities to identify strengths and protective factors that people might derive from their heritage, but it also misses the chance to acknowledge and explore racial trauma, which would give vital context to a person's difficulties, and how they see and experience the world. In addition to informing effective formulation and intervention, enquiring about these experiences might provide the person with an experience of validation that they might not have had previously (Gurpinar‐Morgan et al., 2014). However, there is a level of risk involved if the therapist has little experience or training in handling these conversations with professionalism and cultural humility (Galán et al., 2021; Samuel & Simonds, 2023). This is reflected in some clients' concerns about disclosing racism in therapy due to past experiences of being misunderstood, invalidated, or silenced (Beck, 2019). Furthermore, if racism is directly relevant to someone's mental health and they feel that their therapist is avoiding the subject, this may be taken as evidence that the therapist, too, is racist, or that therapy is not ‘for’ them (Naz et al., 2019).

### Training of psychological professionals

Hemmings and Evans (2018) found that 71% of counsellors had come across racial trauma in their clinical work, yet only 33% reported having had training to identify racial trauma, and just 19% had received training regarding intervention. Other authors have highlighted a lack of attention to cultural humility in therapists' training. Recognised as an important stance in addressing healthcare disparities across a range of helping professions, cultural humility is characterised by a ‘lifelong, active commitment to ongoing learning and self‐reflection in service of building mutually respectful partnerships, stronger working relationships and redressing power imbalances when working interculturally with patients, colleagues and communities’ (Samuel & Simonds, 2023, p. 110). This is underpinned by a practitioner's ability to be: ‘other‐oriented’ (Hook et al., 2013, p. 354) in valuing and respecting the unique identities and experiences of the person in front of them; reflexive; open to new perspectives; aware of, and willing to acknowledge, the limitations in their cultural knowledge; and committed to redressing systemic inequities, power imbalances and social injustices (see Samuel & Simonds, 2023 for a detailed review). Despite endorsement of this stance in the training of medical, social work, counselling and psychotherapy professionals, the training of clinical psychologists and CBT therapists in the UK do not sufficiently focus on the development of skills to work with people from minoritised racial and ethnic groups with cultural humility (Naz et al., 2019). Relatedly, they fail to sufficiently incorporate knowledge of how systemic oppression, such as institutional racism, affects these communities (Galán et al., 2021). Insufficient training in racial trauma and cultural humility perpetuate racialised mental health care disparities, such as reduced access to mental health support, increased early termination of therapy, increased inappropriate diagnosis, and lower treatment satisfaction for racially minoritised groups (Galán et al., 2021). In contrast, studies have found improved therapy outcomes and engagement for people from minoritised racial and ethnic groups when they perceive their therapists to show cultural humility (Mosher et al., 2017; Owen et al., 2016). Clinical psychology trainees and course staff have also reported difficulties in having conversations about race, with trainees reporting that conversations about diversity and multiculturalism lack depth and frequency (Neblett, 2019), and need to be more meaningfully integrated throughout their training courses (Gregus et al., 2020). Acknowledgement of the above issues has led to recommendations that racism, racial trauma and cultural humility be paid additional attention in training curricula for psychological professionals, as well as in research, training beyond academia and the models, theories and methodologies employed by psychological therapists (Galán et al., 2021; Hemmings & Evans, 2018).

### Research context and rationale

The murder of George Floyd in 2020 threw systemic racism into sharp focus on a global scale, and highlighted the ever‐growing potential for racial trauma to be inflicted vicariously, via televised reports, internet‐shared video footage and general public awareness (Bor et al., 2018; Kirkinis et al., 2018). Since then, the prevalence and destructiveness of racism have been further underscored in a myriad of prominent global and national events, including the racism‐ and Islamophobia‐fuelled riots across the UK in the Summer of 2024 (Ahmed, 2024; Olusoga, 2024). It is reasonable to expect that such heightened exposures to racism might lead to an increased likelihood of help‐seeking amongst racially minoritised people (Beck, 2019), given the traumatising impacts highlighted earlier. However, the pervasiveness of racialised disparities in UK mental health care; lack of attention to the needs of racially minoritised clients in therapists' training; and recognition of therapists' potential to enact further harm, engenders the question of how those who disclose racial trauma in therapy experience the process of doing so. Understanding this experience is a necessary precursor to meaningfully developing the training, practice and supervision of psychological professionals to better serve racially minoritised populations, and confronting the neglected issue of racism's impact on psychological distress from a client‐centred, therapy‐focused standpoint. Further, centring the lived experiences of racially minoritised clients serves to disrupt the chronic marginalisation of their voices in research and practice.

Hemmings and Evans (2018) recommended that a study of this nature should explore factors influencing clients' satisfaction/dissatisfaction with therapy, their perceptions of therapists' effectiveness when they have made disclosures of racial trauma, and what they (would) have found helpful in this context. In doing so, it is important to bear in mind that people's experiences may vary considerably depending on whether disclosures were made voluntarily or elicited through therapist questioning, and whether or not disclosures transpired into further discussions of racial trauma during therapy (Beck, 2019; Gurpinar‐Morgan et al., 2014; Naz et al., 2019). The current study was designed to address these issues, and situates itself within the UK context to target the paucity of attention afforded to this subject outside of the USA. The research question asked: how do racially minoritised people experience disclosing/attempting to disclose racial trauma in psychological therapy in the UK?

## METHOD

### Positionality statement

Qualitative methods were utilised to address the research question. As such, our assumptions and positioning in designing and conducting this research need to be stated. We assume that what participants tell us about their experiences of disclosing racial trauma ‘reflects a social reality that needs to be exposed, acknowledged, and understood’ (Willig, 2013, pp. 69–70), and that this reality exists independently of our knowledge or awareness of it (Braun & Clarke, 2022). These ideas have been referred to as ‘critical realism’, a position which asserts that what we know is constrained by our language and culture, and the concepts they give us (Maxwell, 2012), combining a realist ontology with relativist epistemology. Reflexivity was used in an attempt to aid transparency regarding the choices and interpretations made which are inevitably influenced by our own subjectivities. The first author (NS) identifies as a woman of Black British, Caribbean and Pakistani heritage, and is a clinical psychologist by training. Her experiences of working in UK mental health services before and during the 2020 Black Lives Matter protests were foundational to the current research question. The second author (LS) identifies as a White female academic. Both authors are professionally and personally concerned with improving mental health care for marginalised and minoritised communities.

### Design

We chose to elicit participants' experiences and views via an online qualitative survey, rather than through individual interviews. This decision was made because we were focusing on a topic that is recognised to be difficult to discuss; in‐person interviews may have introduced self‐presentation concerns, or concerns that adverse disclosure experiences might be replicated, and limited what participants felt able to disclose. Conversely, qualitative surveys are well‐suited to facilitating disclosures related to sensitive topics by offering a high level of ‘felt anonymity’, as participants are not required to interact directly with the researcher (Braun et al., 2020; Terry & Braun, 2017, p. 19). They are, therefore, less vulnerable to potential social desirability effects than interviews (Braun et al., 2017; Braun & Clarke, 2013; Terry & Braun, 2017). The additional advantage of online survey data collection was that it allowed us to include a larger number of participants than would have been possible with interviews, and therefore inclusion of a greater diversity of voices and experiences, given the research was not funded. Ethical approval was granted by the University of Surrey Ethics Committee.

### Recruitment and data collection procedures

Participants were eligible if they were aged 18 years or over, identified as belonging to a racially minoritised group, and had discussed, or attempted to discuss, racial trauma in therapy. Participants were not eligible if their therapy had been provided outside of the UK, as this study aimed to focus specifically on psychological therapy and discussions of racism within the UK context. No minimum duration in therapy was specified in recognition that participants' therapy journeys were likely to vary according to a range of factors, including their disclosure experience. A recruitment advertisement was shared on Twitter and/or LinkedIn by several organisations and community groups that specialise in supporting racially minoritised people in the UK (Soul Purpose 360 CIC, Black People Talk CIC, Taraki, and Safe Spaces for Black Women). This approach also facilitated snowball sampling, and so the advertisement may have also been shared in other online locations beyond the researchers' awareness. Participants were able to follow a link from the recruitment poster to the survey, enabling them to participate without identifying themselves or their affiliation to specific therapy providers or organisations.

**FIGURE 1:**
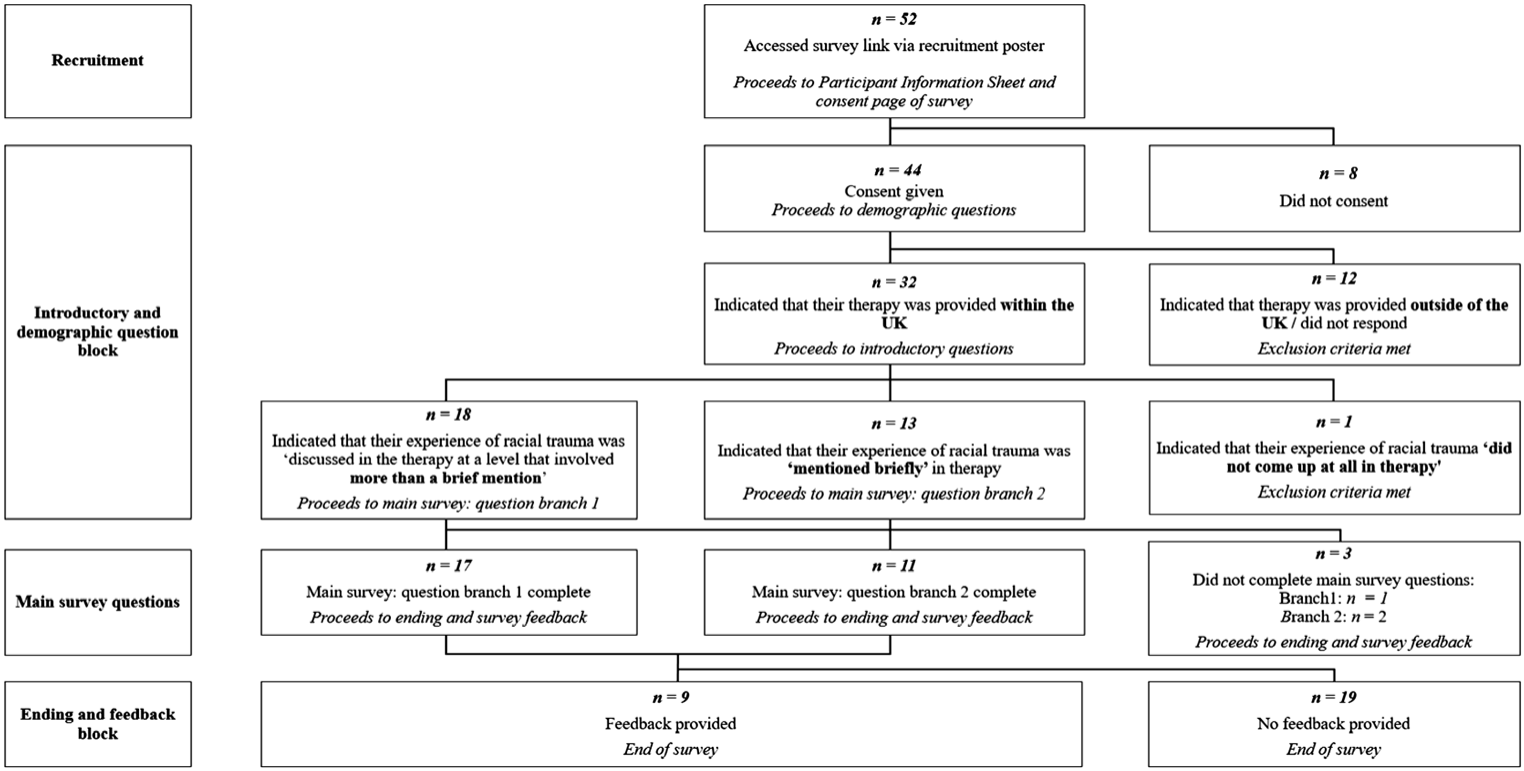
Participant flow through the research procedure.

The data were collected using Qualtrics survey software (Qualtrics, Provo, UT) over a 4‐month period. Survey questions focused on how the subject of racial trauma arose in therapy, and what it was like for participants to talk to their therapist about this. Two slightly different question sets were used depending on whether participants' disclosure of racial trauma had been brief or more substantial. Optional closing questions were included in the survey to invite feedback from participants about their experience of responding to the questions, and of the language and terminology used. This enabled an assessment of the survey's acceptability. Figure 1 shows participant flow through the research procedure.

### Participants

Participant demographic and therapy information is shown in Table 1. A total of 28 eligible participants aged 20–61 (*M =* 35 years; *SD =* 13.11) completed the full survey. Most (*n* = 22) were cisgender women; five were cisgender men, and one self‐identified as genderfluid. All participants were of Black, Asian, or mixed heritage. Although participants were not asked to specify the known/assumed race of their therapists, this information is included as many participants contextualised their survey responses according to racial similarity or difference. Therapy took place in 2011–2022, most commonly in 2020 (nine participants) or later (10 participants). Sixteen participants accessed therapy via a private provider, three via the NHS, three via charities, and five via ‘other’ services (e.g. school, university). Seventeen participants indicated that their experiences of racial trauma were discussed more substantially during therapy; 11 indicated that these experiences were mentioned only briefly and were not discussed further in therapy.

**TABLE 1:** Participant demographic and therapy information (*N* = 28).

Participant demographics			Therapy information				
ID	Racial/ethnic identity	Gender	Year	Modality	Provider	Depth of discussion	Race of therapist^d^
P1	Black – African of Caribbean heritage^a^	Woman or female	2013	Not sure	Charity	More than brief	White
P2	Black – Caribbean	Man or male	2018	Counselling	Charity	More than brief	Not specified
P3	Black – African	Man or male	2015	Not sure	School Therapist	Brief	Not specified
P4	Black – Caribbean	Woman or female	2020	Integrative counselling	Private	More than brief	Black
P5	Black – Caribbean	Woman or female	2021	Person centred	Charity	More than brief	Black
P6	Black – African	Woman or female	2021	CBT	NHS	Brief	White
P7	Indian	Woman or female	2022	Counselling	University	Brief	White
P8	Black British Pakistani^b^	Genderfluid^c^	2020	CBT	NHS	Brief	Not specified
P9	Black – African	Woman or female	2015	CBT	University	Brief	White
P10	White and Black African	Woman or female	2014–2020	Group analysis	Private	Brief	Not specified
P11	Of African descent through enslavement ancestors taken to the Caribbean^a^	Woman or female	2011	Counselling	Private	More than brief	Not specified
P12	Black British^a^	Woman or female	2019	CBT and talking	University and school	More than brief	Not specified
P13	Black Caribbean	Woman or female	2019	Person centred	Private	More than brief	Black
P14	White and Black African	Woman or female	2021^e^	1: Couples therapy 2: Integrative psychotherapy	Private^e^	More than brief^e^	1: Black 2: White
P15	White and Asian	Woman or female	2020	Person‐centred	Private	Brief	Not specified
P16	Indian	Man or male	2016	CBT, MBCT^e^	University counselling services & Outreach Support Social Worker by Mosque and Muslim community^e^	Brief^e^	1: ‘from my ethnicity’ 2: ‘weren't from my ethnicity’^e^
P17	Black – African	Woman or female	2020	Integrative, focus on psychodynamic	Private	More than brief	White
P18	White and Black Caribbean	Woman or female	2020	Group therapy for PTSD	NHS	Brief	Not specified
P19	Black – Caribbean	Woman or female	2020	Mixture of CBT and compassion based therapy	Private	More than brief	Black
P20	Black – African	Man or male	2021	Integrative	Private	More than brief	White
P21	Indian	Woman or female	2021	Integrative counselling	Private	More than brief	White
P22	Black – Caribbean	Woman or female	2021	Person‐centred	Private	Brief	Not specified
P23	Black – African	Woman or female	2020–2021	Talking therapy‐ psychodynamic	Private	More than brief	White
P24	Indian	Woman or female	2017	Not sure	Private	Brief	White
P25	White and Asian	Man or male	2021	Not sure	Private	More than brief	White
P26	Black – African	Woman or female	2022	Gestalt therapy	Private	More than brief	White
P27	Pakistani	Woman or female	2012	Talking therapy CBT, counselling	NHS and work place Occupational Health counselling	More than brief	White
P28	Indian	Woman or female	2020	CBT	Private	More than brief	‘She wasn't white. Perhaps mixed race’

^a^ Participant self‐defined after selecting ‘Any other Black/African/Caribbean background’.

^b^ Participant self‐defined after selecting ‘Any other Mixed/Multiple ethnic background’.

^c^ Participant self‐defined after selecting ‘I use another term/prefer to self‐define’.

^d^ Where available, this represents therapists' racialisation, as assumed by the participant.

^e^ Participant had more than one course of therapy; it is unclear from their responses to which course of therapy these details relate.

### Data analysis

Responses were analysed using thematic analysis (Braun & Clarke, 2006, 2019). NS undertook the initial coding using a primarily inductive orientation and semantic coding to ensure that meanings generated stayed close to participants' language and experiences. To maximise analytic quality, NS used a reflexive log throughout the coding and interpretation process. In addition, LS independently assessed the grounding of the coding and analytic interpretations in the data as an ongoing process in the development of the analytic themes. The analysis was underpinned by qualitative research values, conceptualising ‘researcher subjectivity as a resource for research’ and ‘meaning and knowledge as partial, situated and contextual’ (Braun & Clarke, 2020, p. 39).

Braun and Clarke's (2006, 2019) six stages of thematic analysis are often cited in literature employing an interview approach. Table 2 describes how the analytic process was adapted for the qualitative survey design.

**TABLE 2:** Thematic analysis process.

Phase	Description
Familiarisation	Reading each new/updated response as they were collected and stored by the survey platform. Re‐reading the whole dataset by participant once data collection was complete. Noting ideas related to data items and to the evolving dataset throughout.
Initial coding	Initial coding of data items in survey question order^a^ (i.e. all participants' responses to a single question were coded before moving on to the next). Refined codes by participant, ensuring that they reflected the meanings within each participant's narrative, and to avoid replicating the survey format in the analysis. Capturing important, previously missed meanings as additional codes.
Higher‐level ‘cluster’ coding	Collating and organising all codes into ‘clusters’ according to patterns of similarity and shared concepts to manage high volumes of generated codes. Further refining existing codes and/or noting codes/clusters to be promoted as potential preliminary themes to develop further.
Developing initial themes	Reviewing codes and their respective ‘clusters’. Developing these into potential themes and subthemes, each with preliminary definitions.
Reviewing and naming themes	Iteratively reviewing relationships between themes and how well these appeared to describe the data using thematic maps and reflexive journal entries. Refining theme structures, names and definitions accordingly.
Writing the report	Selecting illustrative data extracts for each theme/subtheme and developing the narrative around these. Making minor changes to theme names/structure as relationships between themes were further illuminated within the write‐up of the finalised analysis.

^a^ This is unlike the coding of interview data, which is typically organised by participant.

## ANALYSIS AND DISCUSSION

### Overview of themes and subthemes

**FIGURE 2:**
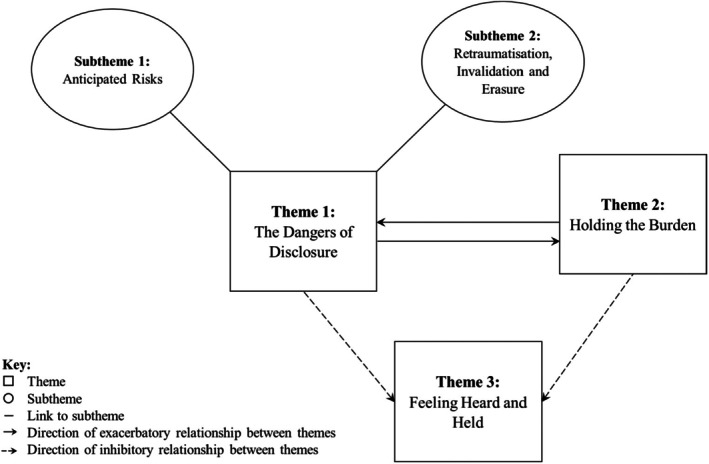
Thematic map showing main themes, subthemes and relationships between them.

Analysis resulted in the development of three interconnected superordinate themes: (1) The Dangers of Disclosure; (2) Holding the Burden; and (3) Feeling Heard and Held (see Figure 2). Participants spoke of the ways in which disclosure felt unsafe, which is captured in ‘The Dangers of Disclosure’. Subthemes include ‘Anticipated Risks’, capturing participants' fears and concerns about the potential experience and/or consequences of disclosure *before* one had occurred, and ‘Retraumatisation, Invalidation and Erasure’, which highlights their experiences of having racial trauma replicated, invalidated, and dismissed by therapists following a disclosure.

‘Holding the Burden’ describes ways that participants were left with unaddressed emotional impacts of racial trauma. These experiences connect to the first theme in two ways. Firstly, due to previous invalidating and dismissive experiences of disclosing racial trauma, some participants were left ‘Holding the Burden’ by having to limit their disclosures to protect themselves from these potential ‘Dangers of Disclosure’; this was exacerbated when therapists did not facilitate deeper discussions as hoped, leaving participants with the additional burden of feeling that their experiences were only superficially explored. Secondly, reflecting the ‘Anticipated Risks’ identified in the first theme, participants were left ‘Holding the Burden’ by being made to feel responsible for initiating discourse about race and racism, educating therapists about racial trauma, and protecting/prioritising therapists' feelings, at the expense of their own needs for containment and validation. These burdensome experiences (further) compromised the safety and usefulness of therapy.

Whilst the previous themes evoke a sense of jeopardised or inhibited safety, understanding and containment relating to participants' disclosures of racial trauma, the third theme, ‘Feeling Heard and Held’, captures the reverse. Participants described ways that their therapists supported them to feel recognised, understood and validated in relation to their experiences of racial trauma.

The themes are discussed further below. Participants' quotes have been edited for typographical and grammatical errors. Text added for clarity are enclosed in square brackets ‘[]’, and omitted data are represented with ‘…’. Quotes have been tabulated to allow more of the participants' voices to be represented within this article, and in order that readers can assess the grounding of the analysis.

### Theme 1: The Dangers of Disclosure

This theme captures the ways in which disclosing racial trauma felt unsafe. Illustrative quotes for this theme and subthemes are presented in Table 3.

**TABLE 3:** Subthemes and illustrative quotes for Theme 1: ‘The Dangers of Disclosure’.

Subtheme	Participant ID	Illustrative quotes
Anticipated risks	P26	White people rarely respond well to these discussions so my expectation was that my therapist would be the same. My therapist is a White male, so I anticipated therapy not being a space where race would be attended to. I had expectations that … racism would have to be named by me. I also expected that I would have to spend time explaining my experiences and why they were harmful.
	P4	I cannot be sure I would have voluntarily brought up race with a counsellor which could not personally relate to my experiences or not be a person of colour. It would feel awkward and [I] would fear I would then have to educate and fight for my experiences to be looked at as valid. … Living in the UK, racism is usually ignored and many people will state that they do not see what people of colour are enduring. This ignorance is othering and victim blaming. There are very real fears that this could be experienced in the therapy room.
	P1	… I didn't trust her because people that looked visibly like her were the cause of me being there. I kept conversation to a minimum and my answers as short as possible. I expected to quit after session 1 because of the same racism
	P24	… I am wary of bringing experiences of racism to a white therapist for fear of dismissal.
	P21	It felt difficult to talk about my feelings … My therapist is of the same demographic as them – white English middle‐class – so I feared he may mistake my account and feelings of the experience as racism or criticism towards him, as I was also sometimes referring to the dominance of white English people in society and white fragility as a backdrop to these conversations. I suppose I feared his feelings – the white English feelings – would take the space and voice and focus away from mine, the ethnic minority space and voice and focus, as does happen in society. I was maybe worried I would be shamed and invalidated.
Retraumatisation, invalidation and erasure	P10	The racial trauma I experienced … flooded back to me in a group full of white people … Often dynamics occurred in the group which replicated the racial trauma I'd experienced … racial expressions such as n‐word or racist jokes would be told and when I reacted to these and tried to explain why I was activated in terms of past racial trauma I would be silenced, mocked, erased, attacked. Never did I manage to get across the depth of racial trauma or how racism shaped and organised my life. My attempts to explain were typically seen as excuses serving to deflect from something else. … I found it degrading and heartbreaking. I still do. … racism was repeated in the group basically and even writing about it now I feel viscerally activated.
	P27	… Based on my experience racism continues to be understood as overt, individual expressions rather than as systemic issue. I felt I needed to justify why I framed my experiences as racism, which left a sense of testimonial injustice, i.e. I was not being believed, I was [thought to be] imagining or reducing my experiences to racism … this only added to the racial trauma and so the therapy sessions themselves became traumatic. … I would not access psychological support again! … It has silenced me, it's been 10 years and I continue to suffer in silence, which is having a physical toll on my health. Therapy perpetuated the racial harm.
	P24	I felt that as usual anything that i may bring up to do with race had been overlooked again, which has been my experience time and time again. I felt like it is not an important issue for my therapist but it [was] a big one for me. … I felt that my therapist did not understand the implication of this incident on my sense of self. I felt as though my feelings were dismissed … .

	P18	I bought up the racism I've experienced in mental health services, and was told it was an inappropriate topic. I wasn't allowed [to discuss my experience further]. I assume to support white people's delicate sensitivities, and to protect statutory services from owning up to their faults. I would have liked to discuss [my experiences] or at the very least have them validated as real trauma … They didn't seem to care/were more interested in other members of the group's trauma … I was kicked out of the group after the session (it was the first session), to make room for someone ‘who would actually benefit’.
	P7	Please do not describe our racist experiences/racial trauma as ‘alleged’ when clients have opened up about such sensitive, personal issues as this can be extremely invalidating, particularly in a society where victims are not believed and it is likely such racism has been downplayed and brushed over before and would feel more hurtful in therapy.
	P13	I specifically chose a Black therapist as I thought it would be a safer space to discuss the issue … [they told me] no one can make you feel what you don't want to feel. I felt belittled. … I got the sense she thought I should be more grateful for my privilege.
P14	… in couples therapy my black therapist insisted that I try to focus on how hard it was for my white partner to not be able to fully relate to my experiences of racism and this was extremely unhelpful. I never wanted to discuss racism in couples therapy again as I felt misunderstood, deprioritised and actually a bit like I needed to somehow bear more because as a black woman I had broader shoulders.

#### Subtheme 1: Anticipated Risks

Many participants anticipated that disclosing experiences of racism would be challenging, and might expose them to further harm. This contributed to a sense of a disclosure being risky and unsafe even before one had been made. This sentiment is comparable to the expectations of further discrimination and stigmatisation associated with disclosure of stigmatised identities (Chaudoir & Fisher, 2010), and supports previous literature relating to racially minoritised people's awareness of the potential social costs of disclosing racism (Garcia et al., 2005; Stangor et al., 2002). All participants who identified such feelings of apprehension and scepticism towards disclosing racial trauma connected these feelings to working with white therapists, with some participants making further connections to their wider experiences of discussing racism with white people (e.g. P26). P4 described ‘very real fears’ that white therapists' responses to racial trauma would mirror wider reticence towards recognising and talking about racism in the UK, and the additional harm this inflicts by invalidating people's lived realities, forcing them to justify their experiences as racism, and to manage its impact in isolation. P4 indicates that invalidating responses would be further influenced by white therapists' lack of lived experience of racism, leaving them unable to understand racial trauma. P1 reflected that her therapist's whiteness signified a possible threat of retraumatisation and managed this by inhibiting her disclosures as a means of protecting herself from further harm, and by holding the prospect of quitting therapy in mind as a means of escaping racism, should it arise.

Participants spoke specifically of concerns that they would be ‘invalidated’ (P4; P21), ‘shamed’ (P21), or dismissed if they attempted to disclose racial trauma to white therapists (P4, P24, P21). Notably, the concerns articulated by these participants and the validity of such fears have been well‐documented by scholars across a number of disciplines (Beck, 2019; Eddo‐Lodge, 2018; Hicks & Butler, 2021; Kinouani, 2021; Lowe, 2014; Patel, 2021). Participants' apprehensions about disclosing to white therapists reflects a wider social context in which doing so is often met with denial, dismissal, minimisation, avoidance and silencing of their experiences, highlighting the challenge of navigating even the *decision* to disclose.

Participants also spoke of concerns that sharing their experiences of racism with white therapists would lead to additional burdens on top of the distress of racial trauma. It is not uncommon for racially minoritised people to encounter ‘cultural naivety’ (Memon et al., 2016, pp. 5–6), to be positioned as ‘educators’ on race and racism (DiAngelo, 2018; Patel, 2021), or to have their experiences overlooked altogether when attempting to disclose experiences of racism. These realities are reflected in the concerns of P26 who expected to not only have to initiate conversations about racism, but to also have to educate her therapist about the harm caused by it.

Participants' concerns about white therapists' possible emotional responses to their disclosures reflected an awareness of the ‘emotional maelstrom’ white people often experience in response to discussions of race and racism, and the possible consequences of this (DiAngelo, 2018; Lowe, 2014, p. 11). P21 reflects this concern, noting the challenge of speaking about racialised societal disparities that underpin her experience of racial trauma whilst privileging white people. These expectations can be understood to demonstrate ways that ‘white fragility’ (P21; DiAngelo, 2018) functions to redirect social resources away from racially minoritised people towards white people. For many participants, their own therapeutic needs had to be balanced with mentally centring their white therapists' possible blindspots and emotional responses to discussions of racism (DiAngelo, 2018), reflecting that ‘Anticipated Risks’ were one way that systemic whiteness and institutionalised racism (Patel, 2021) might operate in therapy.

#### Subtheme 2: Retraumatisation, Invalidation and Erasure

This subtheme captures participants' experiences of invalidation or dismissal upon disclosing racial trauma, as well as occurrences in therapy which mirrored or replicated the experiences of racial trauma for which they had sought support. P10 described her experience of overt racism within a group therapy setting and demonstrates the retraumatisation of experiencing racism in therapy. This leaves her having to contend with the impact of both her immediate and past experiences of racism simultaneously, whilst also being made to justify her distress and to navigate the additional invalidation and ‘attack[s]’ this was met with. The painful experiences of shame, humiliation, and ‘visceral’ activation she describes align with the sequalae associated with trauma responses (Kirkinis et al., 2018) and demonstrates the emotional and physiological impact that this experience had, and continues to have, on her.

Retraumatisation also occurs via invalidation of and dismissiveness towards racially minoritised people's experiences of racism. P27 describes having her disclosures disbelieved and minimised. She connects this invalidation to the therapist's limited understanding of systemic racism, the collective blindness towards this throughout wider society (Eddo‐Lodge, 2018; Kinouani, 2021), the resulting sense of having to justify why her experiences constituted racism, and her consequent distrust and disinclination towards future psychological support. P24 similarly describes how therapist blindspots around race and racism led to her experiences being ‘dismissed’ and ‘overlooked’. Whilst blindspots and being ‘overlooked’ evoke a sense of invalidation by omission or passivity on the part of the therapist, P18 describes a more active form of invalidation in her experience of having her disclosure about racism in services ‘shut down’. Her account conveys a sense that delegitimising her experience and the structural racism that facilitated it (Eddo‐Lodge, 2018) served to shield white group members and the reputation of ‘statutory services’ from the realities of racial trauma, at the expense of her need for support.

Invalidation also occurred via individualistic approaches to racial trauma. P27 described the ‘unhelpful’ and ‘insulting’ experience of her therapist placing the onus on her to learn ‘coping strategies’ and ‘build resilience’ to manage experiences of racism. This account demonstrates how discourses of resilience can individualise and decontextualise distress and locate its cause in the client rather than in systemic oppression (Shwaik, 2023). In this way, therapy may increase distress, leaving clients with the unmitigated impact of racial trauma, as well as implying that suffering is a result of insufficient ‘coping’. This reflects the ways that dominant discourses, therapeutic models and approaches may reproduce and reinforce institutionalised racism, epistemic violence (Patel, 2021) and Eurocentric values and ideas at the detriment of racially minoritised people (Hicks & Butler, 2021; Patel, 2021).

Minimising experiences of racial trauma was another form of invalidation faced in therapy. P7 reflected how therapists' use of language communicates doubt and disbelief about the experiences that have been shared with them. Failing to accept clients' experiences as racism when they have described them as such both implies that perpetrators should be protected, and replicates the wider social burden imposed upon victims of racism to prove their experiences, within a space that should be safe to share sensitive experiences without fear of scrutiny. Minimisation of racial trauma also took the form of therapists providing ‘alternative explanations’ (P27), and evoking notions of naivety and ‘positive intent’ of perpetrators of racist acts (P21). These acts suggest that participants had misinterpreted their experiences as racism and that another explanation would be more suitable/accurate, and trivialise the impact on them by allowing racism to be denied. These therapists' actions parallel ‘the kind of racism that knows better than you, constantly rationalising itself away, weaponising innocence and good intentions’ (Kinouani, 2021, p. 14), and facilitates therapists' avoidance of recognising racism.

Experiences of invalidation occurred with racially similar therapists, as well as white therapists. The safety that P13 had expected in working with a Black therapist was compromised. P14's experience of invalidation from a Black therapist in couples therapy was brought about through the centring of her white partner and invocation of the racialised stereotype of the ‘Strong Black Woman’ (Donovan & West, 2015), both serving to minimise and ‘deprioritise’ her pain.

### Theme 2: Holding the Burden

This theme captures the additional burdens placed upon and navigated by participants following their disclosures of racial trauma. Illustrative quotes are presented in Table 4.

**TABLE 4:** Illustrative quotes for Theme 2: ‘Holding the Burden’.

Participant ID	Illustrative quotes
P8	Any mentions of my experiences of racism impacting my diagnoses weren't discussed in depth. … My first two therapists overlooked this … [my] final therapist looked at this a bit more and in some detail but not completely. I feel like having those conversations more would have helped … to better support me in my therapy journey.
P7	I struggled to continue to talk about it on my own basis … and hoped they would help open up the conversation further but [they] did not. … I would very much have … liked to talk in detail about my experiences as I feel I have bottled them up and had hoped it was in this setting that I could finally talk about them. Sometimes simply giving sympathetic responses is not enough and it is necessary to divulge into the deeper issues and effects.
P15	Because my skin is fair … I do not feel I have experienced the same level of discrimination as my other siblings … which came across as a resistance to discuss this further. … [my therapist] spoke about issues more broadly rather than deepening the experience. I wish my therapist felt more able to push through my conflict. I believe it would have been helpful to validate my experiences … but it never seemed to come up in conversation, which felt like it added to my imposter syndrome. … I wish I felt confident enough to bring it to therapy myself but I do not believe they identified my pain at that moment and that there was more to be explored.
P24	My therapist was a white female. My colleague was also a white female and I was hesitant to bring up the incident. I had felt that maybe if I brought this issue of racism up, my therapist would feel uncomfortable. From the comments made by my therapist, it occurred to me that she just did not get it. I felt that she was empathic but the fact that she did not acknowledge that race was a factor in the incident, made me realise that she did not understand my subjective experience fully. … Whilst I do not refute that empathy is there, that is not enough alone.
P27	There were times that the therapist was visibly uncomfortable upon hearing about the racial injustices that were taking place … I would have to moderate and withhold certain feelings so as not to make the therapist uncomfortable.
P16	I was first allotted someone from my ethnicity who really did a great maintaining transparency. But at other places, I felt disgusted and suffocated where the therapist themselves didn't know how to separate their set of challenges as well as the community's hidden reality. I ended up changing my therapist to someone who does not derive from my culture and [who could] remain impartial as well as openness.
P25	… her responses were somewhat broad brush quotations I'd heard before such as ‘I can't believe this still happens' etc which didn't really add any value and was somewhat frustrating. I feel like my therapist was empathetic but … too quickly focused on condemning racism generally rather than putting the emphasis on my [experience] to explore its impact on me.
P12	I did the work of educating and it made me angry and distressed I felt alone. I went to therapy to unload and I never got the chance to I had to educate.

Participants highlighted the challenges of initiating disclosures of racial trauma without supportive guidance or prompts from their therapists. Rather than facilitating opportunities to validate their racial trauma (Gurpinar‐Morgan et al., 2014), conversations often remained shallow and ‘superficial’ (P27). Some participants had such experiences with multiple therapists, which limited the benefits they were able to derive from therapy (P8). Some were left with a sense of disappointment at having missed out on hoped‐for opportunities to discuss their experiences, and consequently, at having to continue to bear their weight (P7, P15). For P7, the therapist's failure to ‘open up the conversation further’ meant that the emotional impact of racial trauma was missed, and ultimately not understood, despite its significance to the client. P15's therapist had been unable to help her to consider the conflict that she felt in occupying an identity which makes her both a target of racial oppression, and a beneficiary of colourism. This resulted in a missed opportunity to understand and help to ease P15's ‘pain’, and ultimately compounds her distress by leaving her to struggle with the combined impact of racial trauma and heightened sense of fraudulence attached to her experience of this.

Whilst these participants suggest a willingness to tolerate the discomfort necessary to access deeper discussions of their experiences, others noted that discomfort was often located in their therapists. P24 noted ‘I often find that it is uncomfortable for a white therapist to breech the subject’, suggesting that despite the challenges and vulnerability inherent in doing so, clients must often assume greater responsibility for initiating discussions of racism and racial trauma whilst working with white therapists. Anxiety, guilt and discomfort about engaging with race and racism are known issues in the psychological therapy professions (Beck, 2019; Naz et al., 2019; Wood & Patel, 2019), and may support an understanding of why these participants were left holding the responsibility for initiating these discussions. P24 observed that her therapist's race and gender were the same as those of the person who had subjected her to racism, leading her to censor her disclosure to avoid making the therapist feel ‘uncomfortable’. Such conflict about disclosing may serve to protect racially minoritised people from the possibility of white confidants' offence (DiAngelo, 2018). P27 wrote that her therapist's visible discomfort when hearing about racial injustices caused her to withhold her own feelings, resulting in what might be seen as a reversal in client/therapist roles; the participant must contain her therapist's discomfort by suppressing her own feelings. The client is then encumbered not only with the weight of her own trauma, but also the burden of being unable to express this, and of having to hold her therapist's emotional response. The impact of the therapist's response to this disclosure demonstrates the ways that white people's emotional and behavioural responses to racism can ‘block any entry point for reflection and engagement’ with the subject (DiAngelo, 2018, p. 122). P16's account demonstrates that ethnically similar therapists can also burden clients with their response to a disclosure if the therapist centres their own experiences. Together, these examples highlight the necessity for *all* therapists to be aware of their own relationship to issues of racism to effectively manage their personal responses to the subject and remain client‐oriented in their handling of these discussions.

Some participants noted that even when therapists were perceived to demonstrate sympathy/empathy in response to their disclosures, this in itself was ‘not enough’ (P7, P24) if the therapist was unable to also convey an understanding of, or willingness to explore, the effects of racism. Others found that an empathic response which attempted to recognise racism could be undermined by ‘frustrating’, ‘broad brush’ remarks (P25) wherein the therapist resorted to generic condemnation of racism rather than meaningfully recognising the specific impact it had on them. The need to ‘educate’ (P12) therapists about racism and racial trauma further indicated therapists' failure to understand participants' experiences, and created yet another load for participants to bear following disclosure. This mirrors racially minoritised people's experiences in wider society, where it is not uncommon to be expected to explain and volunteer experiences of racism for the benefit of white people (DiAngelo, 2018; Memon et al., 2016; Patel, 2021). Whilst relying on racially minoritised people to educate others on race and racism is problematic (DiAngelo, 2018), it is evident that this tendency was repeated in therapy. P12 further illustrates the additional distress and sense of aloneness associated with having to educate their therapist; the experience ultimately overtakes therapy, leaving their needs unmet.

### Theme 3: Feeling Heard and Held

This theme captures the range of factors that participants identified as contributing to feeling understood and emotionally contained in sharing their experiences of racism with their therapists. Illustrative quotes for this theme are presented in Table 5.

**TABLE 5:** Illustrative quotes for Theme 3: ‘Feeling Heard and Held’.

Participant ID	Illustrative quotes
P28	I felt she got it ‐ she gave me space to explore it and didn't judge me whereas everyone before had defended [the perpetrators] like oh they were just young. It didn't make it right. She heard me for the first time.
P26	Most importantly, he does not try to search for alternative explanations to understand or reframe what I am calling racism. That is so important to me feeling heard, accepted and validated. My therapist was very good at creating a safe atmosphere. He acknowledged his race and cultural background and his awareness of how this might impact me. He invited me to think about this. This then helped me to name more explicit experiences as and when they happened. My therapist would also point out the racial element in interactions, when I did not. My therapist's ability to be reflexive and consider his own racial identity [influenced my decision about whether or not to volunteer these experiences] … . He is also good at acknowledging his limitations but not in a way that makes me feel like I have to educate him or manage his emotions in the process. It was mostly meaningful because my therapist was able to show me that white people can respond helpfully to these experiences. He has been able to model a positive relationship with a white person, a white man and someone who holds considerable power.
P5	… It helped that my therapist was black too, so I felt there was that mutual understanding. I think that also led her to being more open to asking questions [about racial trauma] … which led me to talk about it more. I enjoyed [the conversations we had]. … It helped me feel connected to her and the discussions we had.
P19	My therapist was Black so I found it so valuable that she understood where I was coming from. I didn't have to over‐explain anything. … [I was] completely satisfied. Again, I think this is due to her being Black and having knowledge of racial trauma. I was able to go away and research this more.
P14	My therapist is white, so I asked about her competency with this [helping me to process racism and my racial identity] in our initial contact. … I didn't have any unhelpful experiences with my current individual therapist, owing mostly to our very strong and enduring alliance as well as her level of experience and knowledge … . Although [my therapist] is white, she showed real understanding and empathy without a hint of white guilt, minimisation or other ways of centring herself that usually make talking to white people about racism so difficult and dissatisfying. … White people showing acceptance and resilience in facing the harm of racism makes it possible to share this particular hurt with them, in my experience. … It was important to be held in this type of trauma where there is so very little space to have that experience in this world.
P17	I really appreciated that she didn't make it about her discomfort/feelings about racism as a white person. This really helped because my past experiences of talking to white people about racism have ended up centring the white person. It gave me a chance to be honest about how sad, scared, angry and hurt I felt.

Several participants indicated that feeling heard and understood in relation to racism was hard to come by in wider society, making this a particularly important experience to have with their therapist (P28, P14, P17, P26). P26 highlighted the importance of not being challenged to consider their experience as something other than racism, and how this enabled her to feel ‘heard’. Notably, such examples contrast directly with the accounts contained in ‘Retraumatisation, Invalidation and Erasure’, wherein therapists' tendency to explain away racism had deleterious effects on participants. Being believed was a powerful, and rare, experience.

Therapists' ability to understand the impact of racism without participants having to explain this was also considered important. Again, this contrasts with the sense of having to overexplain and ‘educate’ therapists about racism highlighted in ‘Holding the Burden’. Participants connected this understanding to shared racial identity with their therapist (P5, P19). P19 describes a sense of being understood by their therapist as a result of their shared Blackness, and suggests that this commonality freed them of the potential burden of having to ‘over‐explain’ their experiences. The therapist's Blackness served as an indicator of understanding and shared experience, demonstrating how racial similarity and difference is used to establish who can be trusted to understand racial trauma and racialised experiences (Hicks & Butler, 2021). P19 connected their therapist's Blackness to their ‘knowledge of racial trauma’. This suggests that ‘being Black’ was perceived to deepen the therapist's ‘knowledge’ by giving them an embodied perspective, transcending a purely theoretical understanding of racial trauma. It simultaneously demonstrates that racial trauma is not a concept which is automatically familiar to racially minoritised people; the therapist's lived‐ and conceptual knowledge of racial trauma work together to recognise and legitimise P19's experiences by giving them a name. This then suggests that therapists who are not racially minoritised could similarly validate racial trauma, if they possessed sufficient conceptual knowledge of it. This is consistent with the drive to advocate for meaningful integration of attention to race, racism, and cultural humility within psychology and psychotherapy training courses (Beck, 2019; Lowe, 2014; Naz et al., 2019; Samuel & Simonds, 2023; Wood & Patel, 2019). In keeping with this, P14 attributed her positive experiences of disclosure with a white therapist to her therapist's demonstrable ‘knowledge’ and ‘competency’, combined with their therapeutic alliance.

Reflexivity was another way that therapists could demonstrate understanding of racial trauma. P26 described how, despite her therapist's whiteness (and thus absence of lived experience) making racism a more ‘abstract concept’ for them, the therapist's ability to explicitly recognise their racial and cultural differences and potential impact of these, and to highlight racialised experiences on P26's behalf, facilitated her disclosure and helped her to feel safe and seen. These experiences present a notable contrast to ‘Holding the Burden’, in which responsibility for raising the subject of racism and racial trauma and educating therapists lay with the client. Further contrasts were evident in participants' appreciation of their therapists' reflexivity and transparency about the limits of their competence, without being made to feel burdened by a need to educate the therapist to address or compensate for these limitations (P26).

Participants who had positive experiences with white therapists were particularly grateful for this due to the ways in which this contrasted with previous adverse experiences of attempting to engage with white people on the topic of racism (P14, P17, P26). These participants had often signified their pre−/early‐therapy expectation of ‘another’ unhelpful interaction with a white person about race, before describing ways that their experience of disclosure defied the typical responses that they had become accustomed to. For instance, P14 highlights that decentring whiteness was key to facilitating a rare experience of genuine ‘understanding’, ‘empathy’ and containment by their white therapist without the typical repercussions of dissatisfaction and burdensomeness. P17 similarly indicated that decentring whiteness was an important aspect of their experience of feeling heard and understood when working with white therapists. This enabled her to attend to her own emotional experience without having to simultaneously manage those of a white conversation partner.

## SUMMARY AND FURTHER DISCUSSION

The results were consistent with previous literature demonstrating the range of potential harms that could, and often do, befall racially minoritised people in their attempts to share experiences of racism (Eddo‐Lodge, 2018; Kinouani, 2021; Memon et al., 2016; Patel, 2021). The manifestation of anticipated and actual racial harm in therapy is noteworthy given the well‐documented racial disparities prevalent throughout mental health care (Beck et al., 2019; Bignall et al., 2019; Kapadia et al., 2022), particularly relating to poorer therapy experiences and outcomes faced by racially minoritised people. The participants' accounts offer stark insights into their endurance of invalidating, silencing, retraumatising and burdensome therapist responses to their disclosures of racial trauma. This further evidences such disparities, as well as demonstrating the operations of systemic whiteness and institutionalised racism in UK psychological therapies (Patel, 2021), and the fields of clinical psychology and mental health more broadly (Beck et al., 2019; Bignall et al., 2019; Cummings Centre for the History of Psychology [CCHP, 2023]; Fernando, 2017; Kapadia et al., 2022). Hicks and Butler (2021) note that these systems make it likely that white therapists will reinforce ‘patterns of racial power and White privilege in the therapy room’ (p. 178) when working with racially minoritised clients. The findings therefore call for urgent resolve to safeguard racially minoritised people from racial injustices occurring in the very spaces that they seek to heal from racial trauma.

It is important to also highlight the rarer, and perhaps more surprising, findings which demonstrated that racial harm may also be inflicted by racially minoritised therapists. These findings may, too, be located within an understanding of systemic whiteness and institutionalised racism (Patel, 2021) – by nature of the sociocultural ‘embeddedness’ of these systems (DiAngelo, 2018; Patel, 2021, p. 3), therapists, regardless of race, are unavoidably socialised into the ideologies of whiteness, and are therefore capable of enacting and reinforcing the racism this produces (DiAngelo, 2018; Patel, 2021). This may explain the findings that some racially minoritised therapists still centred whiteness when working with participants' disclosures of racial trauma. That this was possible, but was not expected (i.e. no participants expressed ‘Anticipated Risks’ relating to disclosures with racially minoritised therapists), could speak to the white‐dominated context of psychological therapies; it may have been more difficult to anticipate working with a racially minoritised therapist at all given their underrepresentation within psychological professions (Bawa et al., 2019). It could also reflect how we understand ourselves and others in relation to ‘racial sameness and difference’ (Hicks & Butler, 2021, p. 184); when combined with the concept of ‘racial power’ (p. 178), it makes sense that participants connected white therapists to a potential to inflict harm, and racially similar therapists to an ability to understand and empathise with racial trauma through their own likely experiences of racial oppression. Another possibility is that the UK culture of disinclination to recognise racism as systemic perpetuates a false view of racism as ‘discrete acts committed by individual people, rather than as a complex, interconnected system’ (DiAngelo, 2018, p. 3). This obscures its more subtle operations, and their potential to be reproduced by all, regardless of racial identity; for instance, by centring whiteness and dismissing or overlooking racial trauma. Ultimately, these findings demonstrate that racial retraumatisation, invalidation and erasure can be enacted by all therapists, regardless of race. In doing so, they highlight important issues relating to the dearth of training of psychological professionals around racial trauma (Galán et al., 2021; Hemmings & Evans, 2018; Naz et al., 2019); racial harm in therapy appears not to simply be a case of a few wayward therapists, but a product of the psychological professions' inadequate examination of, and engagement with, whiteness and racism as systems that we are all socialised into (DiAngelo, 2018; Patel, 2021). This enables the reinforcement and reproduction of these systems within our therapy rooms and services, and the training institutions which staff them (Wood & Patel, 2019).

Participants were left with a sense of incompleteness, both in the support they received, and their ability to fully disclose, when required to educate their therapists or protect and prioritise therapists' feelings, whilst their own needs for containment and validation were decentred or unnoticed. This highlights the impact of therapists' blindspots and lack of understanding about race, racism and whiteness, their hesitance to broach or deepen conversations about racism and racial trauma, and inadequate management of their own emotional responses to participants' experiences of racism. Again, participants related these burdens to working with white therapists, reflecting existing literature about the challenges that racially minoritised people experience in talking to white people about racism (Eddo‐Lodge, 2018), and therapists' anxiety about, and avoidance of, engagement with such subjects (Lowe, 2014; Naz et al., 2019; Wood & Patel, 2019). Such experiences contrast with those that resulted in participants ‘Feeling Heard and Held’; in the latter, therapists' abilities to decentre whiteness and demonstrate racial reflexivity and lived‐ and/or conceptual knowledge of racial trauma were key to providing a containing and validating space, freeing participants of the harms and burdens typically associated with such discussions. This has important implications for minimising harm and promoting best practice in psychological work with racial trauma.

### Implications

Whilst stipulations about the need for greater attention to race, racism and cultural humility in psychological therapists' training have largely been proposed by *professionals* in the field (Galán et al., 2021; Naz et al., 2019; Samuel & Simonds, 2023; Wood & Patel, 2019), the accounts of our participants support the case for this from the perspective of *clients'* lived experiences. Though failures to include race and racism in psychological formulations and therapeutic work have been labelled ‘a travesty’ (Hicks & Butler, 2021, p. 179), this continues to be the norm across psychological therapy professions. These omissions will not change unless therapists of all racial identities are able to meaningfully understand and talk about race and racism, acknowledge our complicity in reproducing ‘racist power’ and therefore attend to the ways that whiteness and racism infiltrate clients' lives (Hicks & Butler, 2021, p. 179) and beget racial trauma. Therapists and training providers must take responsibility for their lack of knowledge around racism, racial trauma and antiracist practice through ongoing self‐education and training. Training on these issues should be ongoing and meaningfully integrated throughout curricula, rather than tokenistic (Galán et al., 2021; Samuel & Simonds, 2023). The findings discussed above illustrate the wide‐ranging benefits that could come from active and ongoing therapist education and reflection about racism and racial trauma, including preventing harmful, invalidating and uninformed responses (DiAngelo, 2018) to disclosures of racial trauma; reducing the burdens on racially minoritised clients of not being understood and having to educate their therapists and overexplain their experiences; and promoting meaningful therapeutic support for clients struggling with racial trauma. Targeted training and CPD also have the potential to benefit therapists, supervisors and educators by potentially easing their discomfort or need to come across ‘well’ in having these conversations, minimising the anxiety and avoidance that impede such discussions (Naz et al., 2019; see also Zhu et al., 2023).

The need to increase therapists' racial reflexivity (DiAngelo, 2018; Hicks & Butler, 2021; Patel, 2021) is clear within the findings. Specifically, white therapists in particular need to examine their racial biases; recognise how race and whiteness organises how they see, and are seen by, racially minoritised clients; and take responsibility for their discomfort, defensiveness, and limits in their skill to sensitively address race, racism and racial trauma. This also applies to racially minoritised therapists, who may need to recognise that they may often represent safety and understanding to racially minoritised clients (Hicks & Butler, 2021), but also carry the capacity to do harm if they fail to recognise the ways that they, too, have been socialised into the same systems of whiteness (DiAngelo, 2018; Patel, 2021), and so have the potential to reproduce this. As such, all therapists, regardless of race, need to take an active stance in working against racism, whiteness, and colourism through deliberate self‐reflexivity. Again, this must be coupled with education on whiteness, racism and racial trauma, and should be embedded within the training of psychological professionals (Galán et al., 2021). It is crucial that educators and supervisors undertake their own reflexive work to model and support this process with those they train and supervise (Galán et al., 2021; Patallo, 2019; Samuel & Simonds, 2023; see also Nel et al., 2012). Further, this education and reflexive work must translate into practice; given the inherent power imbalance within the therapeutic relationship, and the challenges of the disclosure process highlighted by participants, therapists should seek to facilitate disclosures of racial trauma such that hoped‐for opportunities for support are not missed, and client burdens are minimised.

Finally, such practice recommendations cannot take place in a vacuum, but must be supported by policy change. Governing bodies overseeing training and service delivery, such as Health Education England (HEE), NHS England (NHSE), the Department of Health and Social Care (DHSC) and modality‐specific professional bodies, must consider how they will facilitate the implementation of such recommendations. This is particularly pertinent as enacting or failing to protect clients from racial harm in therapy contravenes the ethical codes and practice guidelines of many major professional bodies in psychology and psychotherapy, as well as the constitution of the NHS (see Association of Family Therapy [AFT], 2023; British Association for Behavioural and Cognitive Psychotherapies [BABCP], n.d.; British Association for Counselling and Psychotherapy [BACP], 2018; British Psychological Society [BPS], 2017; Department of Health and Social Care, 2021; Health and Care Professions Council [HCPC], 2016, standard 1.5). Actions may include additional funding to develop and maintain antiracism workshops for trainees, supervisors and trainers; and adjusting curricula/accreditation requirements and commissioning targets such that training courses and service providers must evidence their commitment to antiracism, demonstrate outcomes in qualified and trainee therapists' cultural humility, and show meaningful improvements in racially minoritised clients' experiences of psychological therapy. These measures would ensure accountability and facilitate culture shifts away from engagement with race and antiracism as optional add‐ons, towards embedding this as an integral virtue of training and practice. Only if such initiatives are sustained can meaningful change occur.

### Limitations and suggestions for future research

This study has inevitably included the experiences of those who felt enabled to tell their stories of racial trauma disclosure and who, due to the online survey method, were digitally enabled. Whilst ethical considerations were key to this design choice, future research should consider the option of interviews for those who might otherwise be unable to access the study; conducting these via telephone might serve to retain some of the ‘felt anonymity’ (Terry & Braun, 2017, p. 19) that was considered a benefit to the current study's methodology. Furthermore, given the lead author's professional role and social media presence, the recruitment strategy may have led to disproportionate representation of participants with similar professional or educational experiences. As demographic information relating to education and employment was not collected, the authors are limited in their ability to comment further on the transferability or lack of according to these characteristics; however, much of the study's findings support wider literature relating to racially minoritised people's experiences of talking about racism and racial trauma, suggesting that participants' experiences of disclosure in therapy are less likely to be unique to the current sample, as they broadly reflect known racialised social phenomena. Nevertheless, it is recommended that future researchers build relationships with organisations that support racially minoritised communities to sample more broadly. This would also facilitate trust‐building with a population that may be rightfully wary of psychological professionals and researchers, given the history of abuses perpetrated against racially minoritised people within these fields (CCHP, 2023; Fernando, 2017).

It would also be useful for future researchers to collect further demographic information, both to enable greater commentary on the sample's characteristics, and to facilitate considerations of intersectionality (Crenshaw, 1989, 1991) within the disclosure experience, which some participants alluded to. Intersections of race, culture, ethnicity, nationality, and faith may become particularly influential in mediating the disclosure experience and, within racially minoritised client–therapist dyads in particular, similarities and differences across other aspects of social identity might become more salient, and potentially influential, compared to within dyads where the therapist is white. As these identities were not captured in the current study, we cannot meaningfully comment on potential intersectional nuances. Greater attention to this within future research may enable further insight into additional reflexive considerations for therapists to attend to in working holistically with racially minoritised clients.

The current methodology reproduces colonial methods of knowledge‐production wherein decisions are made by researchers positioned as ‘expert’, for a marginalised population who had no say in the design or conduct of the research, whose knowledge and time are extracted (Singh, 2023) with no guarantee of meaningful or tangible benefit to them. Attempts to offset some of these dilemmas threw up other ethical challenges, including the ethics of seeking consultation from racially minoritised experts‐by‐experience or professionals with experience of working with racial trauma, without the ability to offer remuneration or payment. Future research with racially minoritised participant groups should endeavour to coproduce the work with those it is intended to be for and about. Furthermore, we recommend that research‐producing institutions interrogate ethical standards which do not recognise or scrutinise colonial influences on traditional research paradigms, and build relationships with grass‐roots organisations which support and represent racially minoritised populations to facilitate research collaboration and partnership (Singh, 2023). Educators should facilitate this discourse within research methods curricula, as well as promoting participatory action research (PAR) methodologies, such that student researchers learn to engage more critically with research design and ethics. This would help to minimise the oppressive potential of accepted research practices upon marginalised participant populations.

## CONCLUSIONS

This study's findings offer hope in highlighting the positive, healing discussions and responses to disclosure that occurred within therapy, shedding light on what good practice in working therapeutically with racial trauma can look like. The relevance of the findings for all therapy professionals is notable, with therapists from racially minoritised and white backgrounds demonstrating potential to both perpetuate harm and promote meaningful therapeutic support within the context of racial trauma. In this way, our participants' accounts support a shift away from unhelpful assumptions that ‘only’ racially minoritised therapists can work effectively with racial trauma, and that white therapists will ‘only’ perpetuate further harm. Rather, their experiences invite more nuanced conversations which recognise the potential for *all* therapists, regardless of racial heritage, to perpetuate racial harm. Similarly, their experiences highlight the potential for all therapists, regardless of racial heritage, to safeguard against this harm, to offer meaningful therapeutic support for racial trauma, and to develop and demonstrate antiracist therapeutic practice through ongoing education and intentional racial reflexivity. The participants demonstrate that emotional and psychological support for racial trauma is often sought and much desired, challenging the dominant discourse of racially minoritised people being ‘hard to reach’ within the context of psychological therapies (Naz et al., 2019, p. 12), and furthering the ‘counter‐narrative’ presented within other studies of their therapy experiences (Dera, 2021, p. 176). Their accounts also emphasise the need for therapists to take responsibility for facilitating disclosures of racial trauma in order that this support may transpire. Finally, the experiences of our participantsand others who have attempted to disclose racial trauma in therapy, must be amplified to highlight the harms occurring within these spaces, and their contribution to already extensive mental health care disparities faced by racially minoritised people. Therapists, supervisors, service providers, training institutions, and professional bodies must critically examine their commitment to ongoing antiracist therapeutic practice if our professions are to resist further complicity in these inequities. Action must be embedded and sustained systemically within policy, practice and curricula, and within the training and practice of those we are accountable for.

## AUTHOR CONTRIBUTIONS

**Nicole K. S. Samuel:** Conceptualization; methodology; investigation; formal analysis; writing – original draft; writing – review and editing. **Laura M. Simonds:** Conceptualization; formal analysis; methodology; supervision; writing – review and editing.

## CONFLICT OF INTEREST STATEMENT

The authors have no conflict of interest to declare.

## Data Availability

The data that support the findings of this study are available from the corresponding author upon reasonable request.
